# Role of microRNAs and DNA Methyltransferases in Transmitting Induced Genomic Instability between Cell Generations

**DOI:** 10.3389/fpubh.2014.00139

**Published:** 2014-09-15

**Authors:** Katriina Huumonen, Merja Korkalainen, Matti Viluksela, Tapani Lahtinen, Jonne Naarala, Jukka Juutilainen

**Affiliations:** ^1^Department of Environmental Science, University of Eastern Finland, Kuopio, Finland; ^2^Department of Environmental Health, National Institute for Health and Welfare, Kuopio, Finland; ^3^Cancer Center, Kuopio University Hospital, Kuopio, Finland

**Keywords:** epigenetics, cadmium, DNA methyltransferases, induced genomic instability, microRNA, TCDD, cadmium

## Abstract

There is limited understanding of how radiation or chemicals induce genomic instability, and how the instability is epigenetically transmitted to the progeny of exposed cells or organisms. Here, we measured the expression of microRNAs (miRNAs) and DNA methyltransferases (DNMTs) in murine embryonal fibroblasts exposed to ionizing radiation or 2,3,7,8-tetrachlorodibenzo-*p*-dioxin (TCDD), which were previously shown to induce genomic instability in this cell line. Cadmium was used as a reference agent that does not induce genomic instability in our experimental model. Measurements at 8 and 15 days after exposure did not identify any such persistent changes that could be considered as signals transmitting genomic instability to the progeny of exposed cells. However, measurements at 2 days after exposure revealed findings that may reflect initial stages of genomic instability. Changes that were common to TCDD and two doses of radiation (but not to cadmium) included five candidate signature miRNAs and general up-regulation of miRNA expression. Expression of DNMT3a, DNMT3b, and DNMT2 was suppressed by cadmium but not by TCDD or radiation, consistently with the hypothesis that sufficient expression of DNMTs is necessary in the initial phase of induced genomic instability.

## Introduction

Radiation-induced genomic instability (RIGI) is observed as delayed *de novo* appearance of damage (increased cell death, chromosomal aberrations, micronuclei, etc.) in the progeny of irradiated cells or animals (1–3). The mechanisms by which RIGI is initiated and maintained are not fully understood, but they are believed to be epigenetic in nature (4–6). The epigenetic basis is strongly supported by the observed target size (volume that needs to be “hit” by radiation) for RIGI, which appears to be larger than the size of the nucleus (7), indicating that hits on the DNA cannot explain the phenomenon. Ionizing radiation was the first agent that was found to cause this type of instability, but later studies have shown that genomic instability can be induced by other physical and chemical agents as well (8–13).

We use the term “induced genomic instability” (IGI) to describe genomic instability initiated by radiation and other external stressors. Genomic instability, evident as an elevated mutation rate, is one of the characteristic features of cancer cells and is thought to be a driving force in carcinogenesis (14–16). In cancer, genomic instability can result from mutations in genes involved in the maintenance of genome stability (17), in contrast to the apparently epigenetic origin of IGI. However, it is reasonable to assume that IGI and cancer-associated genomic instability represent ends of the same continuum; IGI increases mutation rate in all genes, including those involved in maintenance of genome stability. Research on the mechanisms of IGI is therefore crucial for understanding environmentally induced cancer.

The epigenetic signal that transmits IGI from the exposed cells or animals to subsequent generations is not known. Several recent studies have addressed possible role of changes in microRNA (miRNA) expression and DNA methylation patterns in IGI (5, 18–20). MiRNAs are small non-coding molecules that post-transcriptionally regulate gene expression by binding to their target mRNAs and thereby preventing expression of the target genes. MiRNAs have a role in a variety of cellular processes such as development, differentiation, and growth control (21–25), as well as in several diseases including cancer (26–28). MiRNAs are also involved in epigenetic inheritance (29–31). DNA methylation, involved in epigenetic inheritance as well (32), is probably the most studied epigenetic modification regulating gene expression (33, 34). DNA is methylated on GpC islands where addition of methyl groups is linked to formation of heterochromatin and suppression of gene expression. Effects of DNA methylation on cellular function depend on the sites that are methylated. Global hypomethylation is characteristic of both IGI and cancer, but silencing of particular regions of the genome, e.g., DNA methyltransferases (DNMT) or tumor suppressors, by hypermethylation is often also associated to instability and carcinogenesis, respectively (5, 34–39). Concerning the role of DNA methylation in IGI, two recent studies are of particular interest. These studies showed that functional DNMT, the enzymes responsible for epigenetic methylation of DNA, are necessary for induction of RIGI in mouse embryonic stem cells (40, 41). Global methylation level was not affected by irradiation (41), suggesting a more specific role of DNMTs in IGI. In contrast to the *de novo* methyltransferases DNMT3a and DNMT3b and the maintenance methyltransferase DNMT1, DNMT2 is primarily involved in the methylation of RNA (42). This methyltransferase may also be of interest in relation to IGI, since it has been demonstrated to be necessary for epigenetic inheritance mediated by small non-coding RNAs (29).

Most studies have measured miRNA expression and methylation-related changes only once and relatively soon after exposure. Therefore, it is not possible to conclude whether these changes represent the epigenetic signals that transmit and maintain IGI, or just reversible responses to exposures. In the present study, miRNA expression and expression of DNMTs were assessed in cultured murine embryonic C3H/10T1/2 fibroblasts shortly after exposure (2 days) and at a delayed time points (8 and 15 days) representing IGI that persists in the progeny of the exposed cells. Three different exposures, with known differences in the ability to induce genomic instability, were employed to allow identifying changes characteristic of IGI. Ionizing radiation is genotoxic and has been shown to induce genomic instability in numerous studies. Cadmium is also genotoxic, but did not result in IGI in our previous experiments (8). The dioxin model compound 2,3,7,8-tetrachlorodibenzo-*p*-dioxin (TCDD) is a non-genotoxic carcinogen (43, 44) but appears to induce genomic instability (8). Direct genotoxicity of ionizing radiation and cadmium, lack of direct genotoxicity of TCDD, IGI initiation by ionizing radiation and TCDD, as well as lack of IGI from exposure to cadmium were all shown in our previous experiments with C3H/10T1/2 cells (8, 45). In these studies, direct genotoxicity was measured 2 days after exposure, and IGI was assessed by measuring micronuclei at 8 days (and 15 days for radiation) after exposure. The cell line, doses, and measurement time points used in the present study are identical to those used in the previous experiments.

The analysis of miRNA data included assessment of the heterogeneity of miRNA expression. Increased heterogeneity of gene expression has been previously described multiple generations after irradiation in cultured cells (46) and in *C. elegans* nematodes (47), suggesting that gene expression changes in IGI are a chaotic response of a complex system rather than a specific response of a limited number of genes. Here, we provide data suggesting a similar phenomenon also in cells exposed to TCDD (but not in cadmium-exposed cells). Therefore, as increased heterogeneity of gene expression seems to be characteristic of IGI, it is of interest to study whether IGI is associated with similar non-specific and apparently chaotic changes also in miRNA expression.

## Materials and Methods

### Chemicals

2,3,7,8-Tetrachlorodibenzo-*p*-dioxin was purchased from Ufa-Institute (Ufa, Russia) over 99% pure. The purity was assessed by gas chromatography-mass spectrometry. Cadmium chloride (Fluka, over 99% pure) was obtained from Sigma. Media, serum, and other products for cell culturing were supplied by Gibco (Invitrogen, Paisley, UK).

### Cell culture

Mouse embryonic fibroblasts (C3H10T1/2 clone 8 from American Type Culture Collection) were grown in basal eagle medium supplemented with 10% fetal bovine serum, 2 mM l-glutamine, and antibiotics (100 U/ml penicillin, 100 μg/ml streptomycin) at 37°C in a humidified atmosphere of 5% CO_2_ in air. For plating cells were harvested by 0.25% trypsin (Invitrogen, Carlsbad, CA, USA) in 0.02% EDTA in PBS (w/o Ca^2+^, Mg^2+^).

### Exposure

#### TCDD and cadmium

Cells were plated at the density of 5000 cells/cm^2^. The day after, medium was replaced with medium containing 10 nM TCDD dissolved in DMSO, DMSO vehicle alone (final concentration of DMSO 0.1%), or 1 μM cadmium chloride (CdCl_2_). After 2 days, the exposure medium was removed and cells comprising 2-day samples were harvested. For 8- or 15-day samples, the cells were subcultured and cultured for 6 or 13 more days without exposure.

#### Ionizing radiation

The plated cell density was selected based on the X-ray dose, endpoint, and timing of the measurements. Doubling time of 24 h was assumed [actual time being 16 h with some delay after the plating (48)]. Both for miRNA and DNMT analyses 2 days after exposure, the cell density plated 24 h prior to exposure was ~2700 cells/cm^2^ for the control and the 1 Gy dose. For the 5 Gy dose, the plated cell density was ~4500 cells/cm^2^. For the same analyses 8 days after exposure, the cell densities were ~350 cells/cm^2^ for the control and the 1 Gy dose, and ~700 cells/cm^2^ for the 5 Gy dose. These cells were subcultured 5 days after exposure at cell density of ~2700 cells/cm^2^ for control and 1 Gy samples and at ~4500 cells/cm^2^ for 5 Gy samples. For samples to be collected 15 days after exposure, the cell density plated at this point (8 days after exposure) was ~350 cells/cm^2^ for the control and 1 Gy dose, and ~550 cells/cm^2^ for the 5 Gy dose.

Cells were exposed to X-radiation with a 4 MeV Varian 600C (Palo Alto, CA, USA) linear accelerator in Kuopio University Hospital. Cells were placed on the treatment couch between two plexiglass sheets with thicknesses of 1 cm (above) and 2 cm (below). The control cells were also taken to the hospital facilities. The doses applied were 1 and 5 Gy, the average energy of photons was 1.5 MeV, and the dose rate was ~2.5 Gy/min.

### miRNA PCR array analysis

Total RNA was first isolated from pelleted cells using Trizol reagent (Invitrogen Life Technologies, Carlsbad, CA, USA) and miRNA fraction was purified using RT^2^ qPCR-Grade miRNA isolation kit (SABiosciences, a Qiagen Company) in the first experiment. In the following experiments, miRNA was isolated using miRNeasy Mini Kit (Qiagen, Hilden, Germany) and enriched by RNeasy MinElute Cleanup Kit (Qiagen). Two hundred nanograms of miRNA was converted to cDNA using RT^2^ miRNA First Strand Kit (SABiosciences). cDNA samples were mixed with RT^2^ qPCR Master Mix (SABiosciences) and distributed in every well of a PCR array plate (Mouse miFinder RT^2^ miRNA PCR array by SABiosciences) profiling the expression of 88 most abundantly expressed and best characterized miRNA sequences in the mouse genome. The array plate contained also four housekeeping assays for normalizing the qPCR array data as well as duplicate controls for reverse transcription reaction and for the efficiency of PCR reaction. Applied Biosystems 7000 Real-Time PCR System (Applied Biosystems, Foster City, CA, USA) was used to determine Ct-values of each well. The fold changes in Ct-values were calculated using the web-based data analysis program of SABiosciences.

### mRNA expression of DNA methyltransferases

RNA was isolated from pelleted cells using E.Z.N.A Total RNA Kit and RNase-free DNase set (Omega Biotek, Doraville, GA, USA) or RNeasy Mini Kit and RNase-free DNase (Qiagen). cDNA was generated by Omniscript RT Kit (Qiagen) using random hexamers (Roche) and used as a template for quantitative PCR analysis. The expression levels of DNMT were analyzed using Power SYBR Green PCR Master Mix and Applied Biosystems 7000 Real-Time PCR System (Applied Biosystems). Standard curves were generated using isolated and purified PCR products produced with the same primers designed for quantitative PCR. PCR products were purified from agarose gels using QIAquick PCR Purification Kit (Qiagen) and the concentrations were determined spectrophotometrically using Nano Drop (Thermo Scientific, Wilmington, DE, USA).

PCR primers were designed using Primer Express software from Applied Biosystems that allowed the using of universal thermal cycling parameters. The following primers were used: DNMT1, tgtggatgaaccccagatgtt and tgaacctatgcatgggagaatctt; DNMT2, actgcgatatttcacaccgaaa and gcagccggtaacgctgttt; DNMT3a, gctcaggcagccattaagga and ggagtcgagaaggccagtctt; DNMT3b, tcgctgtgggaactgttaagc and cgggcaggattgacgttaga; GAPDH, gtatgactccactcacggcaaa and ggtctcgctcctggaagatg. PCR reaction was initiated with an incubation step of 10 min at 95°C to activate AmpliTaq Gold DNA Polymerase. This was followed by 40 cycles of denaturation at 95°C for 15 s and annealing/extension at 60°C for 1 min. Dissociation curve was run to confirm the absence of non-specific amplification. Negative controls were included in each run. The expression levels were related to mRNA concentrations of housekeeping gene GAPDH to normalize the amount of cDNA in PCR reactions.

### Gene expression

The expression of 84 genes involved in transformation and tumorigenesis was profiled with the Mouse Cancer Pathway Finder™ RT2 Profiler PCR Array (SABiosciences, a Qiagen Company) as described earlier (8).

### Global methylation

DNA was isolated from pelleted cells using GenElute Mammalian Genomic DNA Miniprep Kit (Sigma-Aldrich, St. Louis, MO, USA) and the concentration was determined using Nano Drop (Thermo Scientific). The relative levels of methylated DNA were measured from 200 ng of DNA using Imprint Methylated DNA Quantification Kit (Sigma-Aldrich) or Methylamp Global DNA Methylation Quantification Ultra Kit (Epigentek, Brooklyn, NY, USA).

### Data analysis

Statistical analyses were performed on DNMT data by ANOVA followed by the Least Significant Difference test (IBM SPSS Statistics 20). In the case of non-homogeneous variances, the Mann–Whitney test was used. The limit of statistically significant difference was set at *p* < 0.05.

## Results

### Changes in miRNA expression

**Figure 1:**
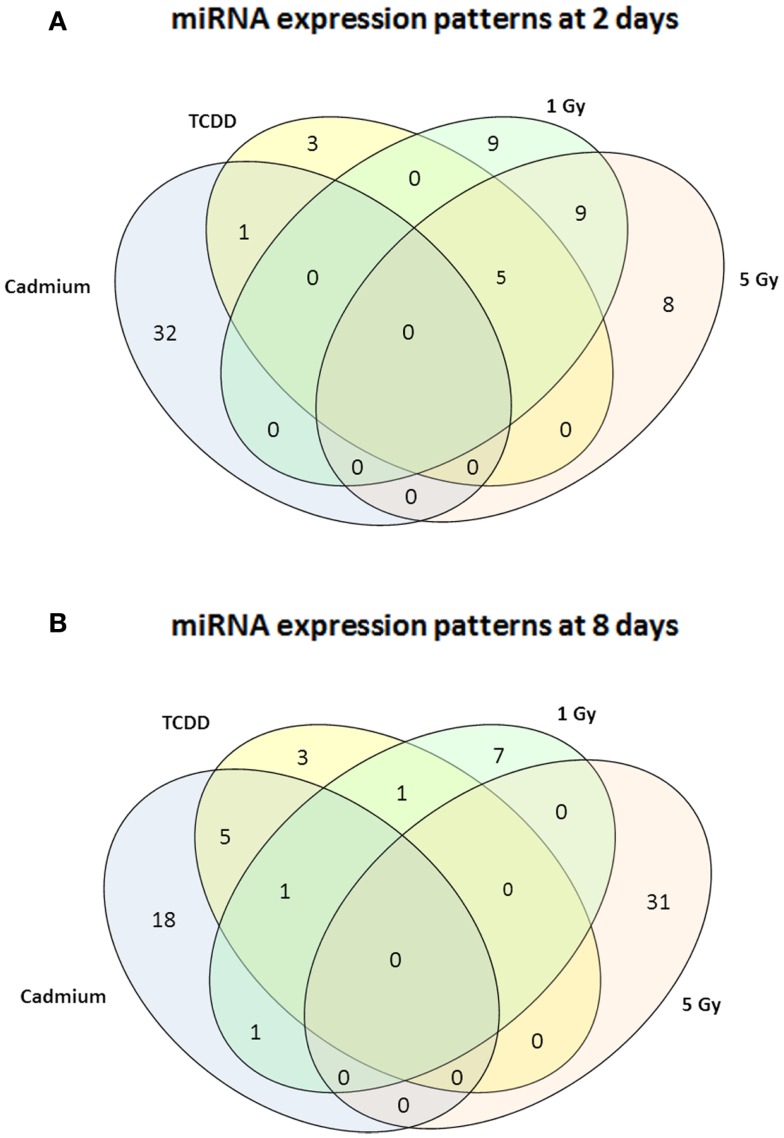
**A Venn diagram showing the number of miRNA – species with altered expression at 2 days (A) or 8 days (B)**. Greater than or equal to 1.50-fold changes in the same direction are presented as common changes.

Murine embryonal fibroblasts were exposed to 1 or 5 Gy of ionizing radiation at a dose rate of 2.5 Gy/min, or for 2 days to 10 nM TCDD or 1 μM cadmium. These treatments with ionizing radiation and TCDD were previously shown to induce genomic instability in this cell line, whereas cadmium was used as a reference agent that did not induce genomic instability in our experimental model. Measurements of 88 miRNA species were performed 2 and 8 days after the treatments. All miRNA data are presented in Table S1 in Supplementary Material. The number of exposure-related changes (consistent direction of change in two independent experiments, average difference between exposed and control cells ≥1.50-fold) in miRNA species is shown in Figure 1. At 2 days (Figure 1A), 23 miRNA species were deregulated by 1 Gy and 22 species by 5 Gy of ionizing radiation. The majority of the changes were up-regulations, 16 at 1 Gy dose and 15 at 5 Gy dose (data shown in Table S1 in Supplementary Material). Fourteen miRNA species were affected by irradiation at both 1 and 5 Grays. In TCDD-exposed cells, 9 miRNAs were deregulated (8 up-regulated, 1 down-regulated). Cadmium-exposed cells responded with changes in 33 miRNAs, from which most were down-regulated (29 down-regulated, 4 up-regulated). The cadmium-induced changes differed from those induced by the other exposures, showing only one common change with TCDD and none with radiation. There were five miRNA species that were similarly affected by both doses of ionizing radiation and by TCDD, i.e., by all exposures that have IGI in this cell line in our experiments (Table 1). All these changes were up-regulations.

**Table 1:** **MicroRNA changes common to TCDD and two doses of ionizing radiation at 2 days after exposure**.

	TCDD	1 Gy	5 Gy
miR-29b	1.8	4.7	2.9
miR-31	1.7	1.7	1.7
miR-101a	1.7	1.8	2.9
miR-130a	1.5	4.2	4.4
miR-199a-5p	1.5	1.9	1.9

*Fold differences between exposed and control cultures are given*.

At 8 days after irradiation at doses of 1 or 5 Gy, 10 (7 down-regulated, 3 up-regulated) and 31 (all up-regulated) changed miRNAs were observed, respectively (Figure 1B). At the same time point, 10 miRNAs were down-regulated by TCDD and 25 miRNAs were affected (all but one down-regulated) by cadmium. Changes common to radiation and TCDD were not detected at 8 days. Six of the cadmium-related changes were common with TCDD. None of the measured miRNA species was affected by TCDD at both 2 and 8 days, whereas nine miRNAs were suppressed by cadmium at both 2 and 8 days.

In case of ionizing radiation, follow-up of miRNA expression was continued until 15 days after the exposure. At this point, 1 miRNA was down-regulated by 1 Gy and 28 miRNAs were affected by 5 Gy (22 up-regulated, 6 down-regulated). There were no miRNA species constantly affected at all time points in the 1 Gy group. In the 5 Gy group, miR-1 and miR-146a were induced at 2, 8, and 15 days, whereas miR-141 was induced at 2 and 8 days, but suppressed at 15 days. Nine miRNA species started showing up-regulation at 8 days and continued to be up-regulated at 15 days.

### Effects on DNA methyltransferases and global methylation

**Figure 2:**
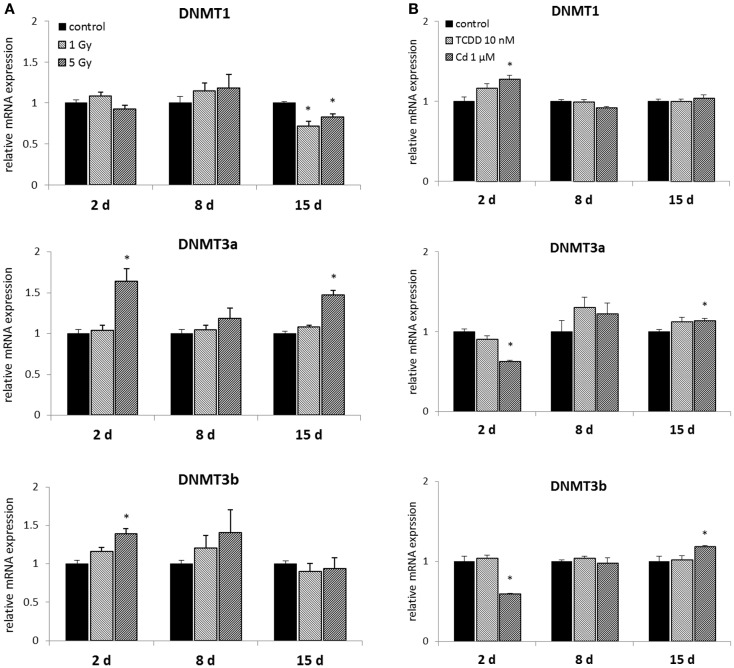
**Direct (2 days) or delayed (8 and 15 days) mRNA expression of DNMT1, DNMT3a, and DNMT3b after (A) exposure to a single dose of 1 or 5 Gy ionizing radiation (*n* = 5 for 2 and 8 days, *n* = 3 for 15 days) or (B) 2 days of exposure to 10 nM TCDD or 1 μM cadmium (*n* = 3)**. An asterisk (*) represents a *p*-value <0.05.

Murine embryonal fibroblasts were exposed to a single dose of 1 or 5 Gy of ionizing radiation, or for 2 days to 10 nM TCDD or 1 μM cadmium. Measurements of the mRNA expression of the methyltransferases DNMT1, DNMT3A, DNMT3B, and DNMT2 were carried out at 2, 8, and 15 days after the treatments. Of the DNA methylating DNMTs, DNMT1 was suppressed by ionizing radiation (both at 1 and 5 Gy doses) at 15 days, but not at 2 or 8 days (Figure 2A). The 1 Gy dose had no effects on the expression of DNMT3A or DNMT3B, whereas 5 Gy of radiation-induced DNMT3A at 2 and 15 days and DNMT3B at 2 days.

2,3,7,8-Tetrachlorodibenzo-*p*-dioxin had no effects on the expression of DNA methylating DNMTs at any of the three time points (Figure 2B). Cadmium, however, significantly affected the expression of all the three DNMTs determined immediately after 2 days of exposure. These changes involved increased expression of DNMT1, and reduced expression of DNMT3a and DNMT3b. No statistically significant differences between cadmium-exposed and control cells were observed at 8 days, but induction of DNMT3a and DNMT3b was detected at 15 days.

**Figure 3:**
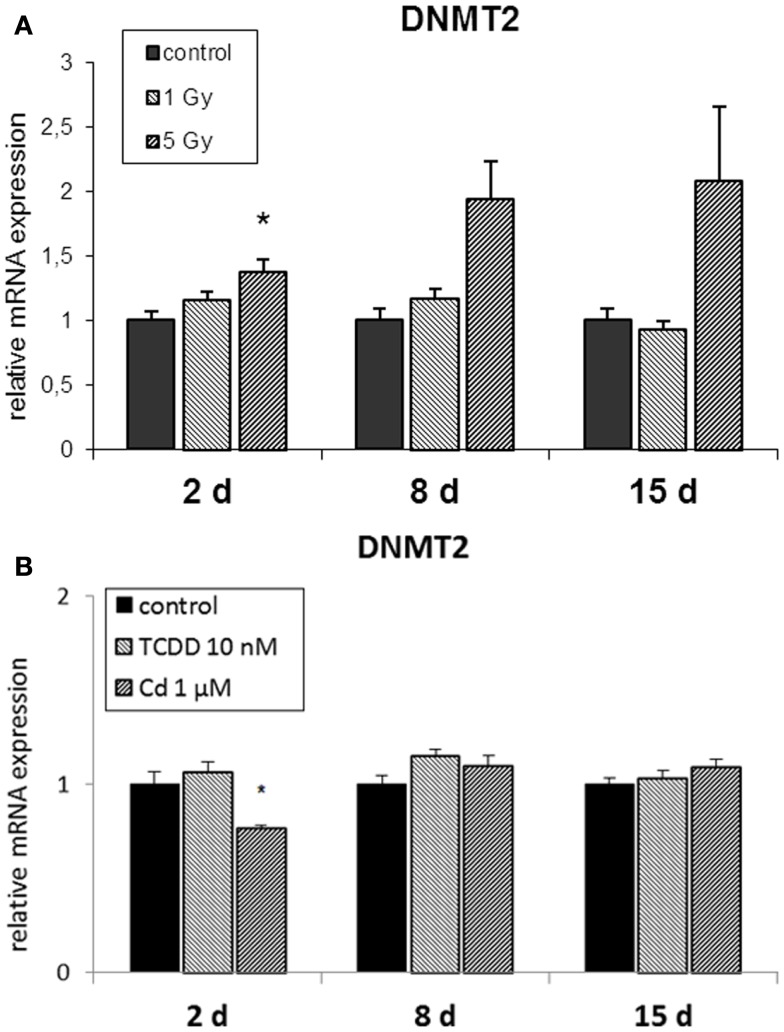
**Direct (2 days) or delayed (8 and 15 days) mRNA expression of DNMT2 after 2 days of exposure to (A) 10 nM TCDD or 1 μM cadmium (*n* = 3) and (B) single dose of 1 or 5 Gy ionizing radiation (*n* = 5 or 3)**. An asterisk (*) represents a *p*-value <0.05.

The RNA methyltransferase DNMT2 was not affected by radiation at 1 Gy (Figure 3A) but it was up-regulated at 2 and 8 days in cells irradiated at 5 Gy. TCDD exposure did not induce changes in the expression of DNMT2, whereas cadmium exposure resulted in its suppression at the end of the 2-day exposure but not at 8 or 15 days (Figure 3B).

We did not detect any consistent changes in the global methylation levels measured by the two different analysis kits used (data not shown).

### Heterogeneity of gene expression and miRNA expression in exposed cells

**Figure 4:**
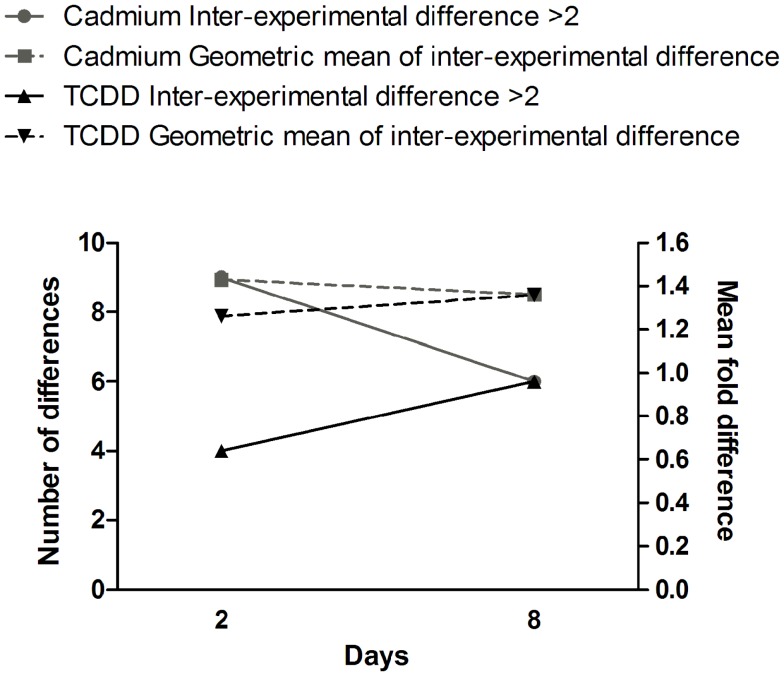
**Time dependence of gene expression differences in cells exposed to cadmium or TCDD**. The left *y*-axis is for the number of differences (≥twofold) between the two experiments (inter-experimental difference). The right *y*-axis is for geometric mean of fold differences between the two experiments.

**Figure 5:**
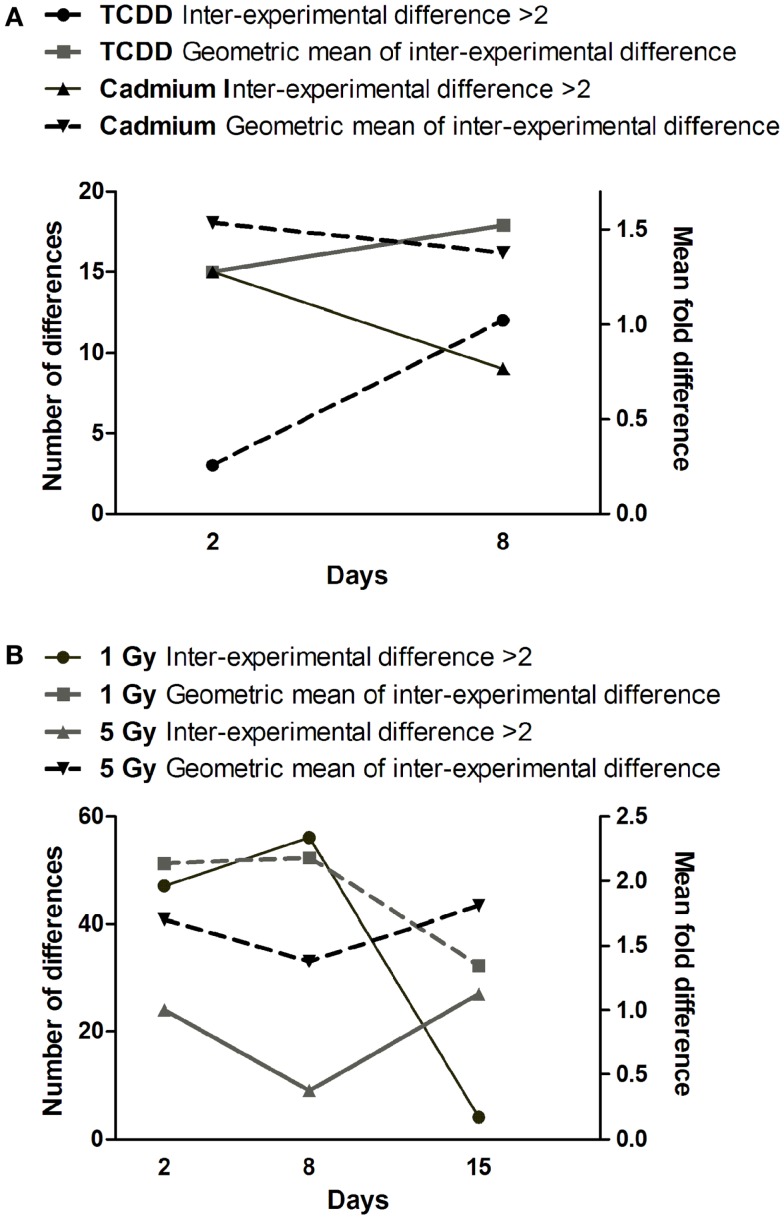
**Time dependence of miRNA expression differences in cells exposed to (A) cadmium or TCDD or (B) radiation**. The left *y*-axis is for the number of differences (≥twofold) between the two experiments (inter-experimental difference). The right *y*-axis is for geometric mean of fold differences between the two experiments.

Comparison of gene expression changes in two independent experiments (Figure 4) revealed that the heterogeneity between the experiments increased with time in TCDD-exposed cells, but a decreasing trend was observed in cadmium-exposed cells. The finding in TCDD-exposed cells is similar to the increased heterogeneity of gene expression observed in the progeny of radiation-exposed nematodes (47) and cultured mammalian cells (46). In the present study, we addressed heterogeneity also in miRNA expression changes. Between days 2 and 8, a trend of increasing heterogeneity was observed only in TCDD-exposed cells (Figure 5A). In cells exposed to 1 Gy of radiation, there was an increase in the number of inter-experiment differences between days 2 and 8, but this trend was not supported by the mean fold difference between experiments, which was almost constant from day 2 to day 8 (Figure 5B). Furthermore, the heterogeneity strongly decreased between days 8 and 15 in the 1 Gy group, reflecting the fact that there were only few miRNA expression changes that remained at 15 days. The findings in the 5 Gy group were quite different from those of the 1 Gy group; the measures of heterogeneity decreased between days 2 and 8, and then showed an increase at day 15 (Figure 5B). Overall, the results did not provide evidence that increased heterogeneity of miRNA expression (similar to that observed for gene expression in several studies) would be characteristic in the progeny of cells exposed to agents that induce genomic instability. However, only 88 miRNA species were measured in two independent experiments; more sensitive experimental approaches might detect increased heterogeneity associated with IGI.

## Discussion

### Is there a specific miRNA signature of induced genomic instability?

In the present study, five miRNAs were similarly induced at 2 days after treatment with TCDD and both doses of ionizing radiation (Table 1), exposures that were previously shown to induce genomic instability in the same experimental set-up. None of these miRNAs were induced by cadmium, an agent that did not induce genomic instability in our previous experiments. The five changes common to TCDD and radiation may, therefore, represent a miRNA signature of IGI. However, this possible signature was detected only at 2 days after exposure and not at 8 days, indicating that these miRNA changes do not represent a signature characteristic of transmission or maintenance of IGI. The five common changes detected at 2 days are nevertheless potentially interesting, as they may represent miRNAs participating in the induction of IGI.

All the five candidate signature miRNAs (miR-29b, miR-31, miR-101a, miR-130a, miR-199a-5p) have been reported to show altered expression levels in different types of cancers (49–58). The Mir-29 family targets DNMT3a and DNMT3b directly, and DNMT1 indirectly (59, 60). Up-regulation of miR-29b can therefore be associated with hypomethylation globally (59, 61) or at specific regions of the genome (19). In our study, miR-29b was up-regulated at 2 days after exposures to TCDD and ionizing radiation, but down-regulation of DNMTs was not detected at the same time point or at 8 days (but transient down-regulation cannot be excluded because of the limited sampling times). Mir-101a targets cFOS, EZH2, and COX-2 (62). EZH2 is a histone methyltransferase that catalyzes methylation of H3 Lysine 27 leading to repression of gene expression (63). In addition, miR-101 has been shown to cause aberrant DNA methylation in hepatocellular carcinoma tissue by targeting DNMT3a (64). Despite the up-regulation of miR-101a at 2 days, we did not observe suppression of DNMT3a expression after irradiation or TCDD –exposure, but instead an induction at 2 and 15 days after irradiation (at 5 Gy). MiR-31 can act both as a tumor suppressor and onco-miR (56). Multiple targets of miR-31 have been identified, including EZH2 (65) and dicer (66). In colorectal adenocarcinoma, overexpression of mir-31 was associated with high level of microsatellite instability (67). MiR-130a also targets dicer1 (68).

The exposures inducing genomic instability (1, 5 Gy, TCDD) shared a common characteristic that the majority of miRNA changes were up-regulations at 2 days after exposure, in contrast to the preponderance of down-regulations in cells exposed to cadmium. The majority of up-regulations in radiation- and TCDD-exposed cells were observed only at 2 days after exposure, so (if it is characteristic to IGI) it might be a signature of induction rather than maintenance of IGI. The prevailing up-regulation of miRNAs after exposure to ionizing radiation has been reported in irradiated mouse testis by Tamminga et al. (69), in thymus of the progeny of irradiated male mice (19, 70) and in human peripheral blood cells (71). Jaksik et al. (72) reported up-regulation of miRNAs in human K562, Me45, and HCT116 cells at 1 h but not at 12 h after irradiation, whereas, e.g., Kraemer et al. (70) found that the majority of deregulated miRNAs were down-regulated at 4 and 24 h after irradiation. As expected, changes in miRNA expression seem to vary as a function of time and depend on the type and dose of radiation, and target organism. It should also be noted that apart from Tamminga et al. (69) and Filkowski et al. (19), these studies did not address the presence of IGI. Therefore, it is not possible to conclude whether changes in miRNA expression were associated with IGI or just irradiation itself.

The number of studies conducted on TCDD- or cadmium-induced miRNA changes in mammalian cells or *in vivo* is limited. Singh et al. (73) found more miRNAs down- than up-regulated (>1.5-fold) in murine fetal thymocytes after prenatal exposure to TCDD. Moffat et al. (74) reported few changes in miRNA-profiles of rat or mouse livers or hepatoma cell lines after TCDD treatment. As for cadmium, Fabbri et al. (75) treated HepG2 cells with 10 μM cadmium for 24 h and measured miRNA expression using a low density array. All of the 12 changed miRNAs were down-regulations.

### Changes in methyltransferase expression and global methylation

Ionizing radiation induced expression of DNMT2 (significant at 2 and 8 days), DNMT3a (significant at 2 and 15 days), and DNMT3b (significant at 2 days). These changes were observed only at 5 Gy dose. However, both doses of radiation resulted in decreased DNMT1 expression at 15 days after exposure. This might be linked to the reported global hypomethylation in IGI (5, 35). However, no changes in global methylation level were observed with the methods used. Of course, this does not exclude changes in the methylation of particular regions of the genome.

2,3,7,8-Tetrachlorodibenzo-*p*-dioxin was not found to cause any changes in DNMT expression. In particular, there was no evidence of decreased DNMT1 expression at 15 days, indicating that such a change is specific for ionizing radiation rather than a general characteristic of IGI.

Cadmium affected the DNMT status directly after exposure as indicated by decreased mRNA levels of the *de novo* DNMTs 3a and 3b. Depending on the experimental set-up, both increase and decrease in the expression of DNMTs have been reported by others (76–78). There is evidence that functional DNMT1, DNMT3a, and DNMT3b are essential for IGI. IGI was not transmitted to the progeny of irradiated cells or to bystander cells, if these DNMTs were not functional in the cells (40, 41). The essential role of DNMTs in the induction of genomic instability is in accordance with our observation that DNMT3a and DNMT3b were suppressed by cadmium (the only exposure that did not induce genomic instability in our experiments), while the expression of these DNMTs was either unchanged (TCDD, radiation at 1 Gy) or increased (radiation at 5 Gy) by the exposures that did induce genomic instability. The expression of DNMTs was suppressed only at 2 days after exposure, while normal DNMT levels were observed at the later time points. This is consistent with the observation that transient suppression of DNMT1 in the irradiated cells was sufficient for preventing the transmission of IGI to bystander cells (40). In our study, cadmium was also the only exposure that suppressed the RNA methyltransferase DNMT2, while a normal or increased level of DNMT2 was observed after the treatments that did induce genomic instability. From this perspective, it is of interest that DNMT2 has been shown to be required for epigenetic inheritance mediated by small non-coding RNAs (29).

### Conclusion

Measurements of 88 well characterized and abundant miRNA species and 4 DNMTs did not reveal any miRNA signature specific to transmitting and maintaining IGI. However, measurements at 2 days after exposure revealed findings that may reflect initial stages of genomic instability. Five miRNA species characteristic of epigenetic regulation were similarly changed at 2 days after exposure to TCDD and two doses of ionizing radiation, but no common changes were observed between these exposures and cadmium. Up-regulation of miRNAs at 2 days was also characteristic of cells exposed to TCDD and both doses of ionizing radiation, while the majority of changes in cadmium-exposed cells were down-regulations. Cadmium was also the only exposure that suppressed the expression of DNMT3a, DNMT3b, and DNMT2 at 2 days after exposure. This finding fits with recent data from other studies, indicating that methyltransferases are essential in the initiation of IGI. Increased heterogeneity of miRNA expression, similar to that observed for gene expression, was not found to be characteristic to agents causing IGI. Further studies would be useful to determine whether the characteristics of initial responses to radiation and TCDD (common miRNA changes, up-regulation of miRNAs, expression of DNMTs not suppressed) are reproducible and common to other agents that induce genomic instability.

## Conflict of Interest Statement

The authors declare that the research was conducted in the absence of any commercial or financial relationships that could be construed as a potential conflict of interest.

## Supplementary Material

The Supplementary Material for this article can be found online at http://www.frontiersin.org/Journal/10.3389/fpubh.2014.00139/abstract
