# Supplementary material for: Role of microRNAs and DNA Methyltransferases in Transmitting Induced Genomic Instability between Cell Generations
Source: Front Public Health. 2014 Sep 15;2:139. doi: 10.3389/fpubh.2014.00139 (PMC4163984; doi:10.3389/fpubh.2014.00139)
Supplement: Supplementary file 1 [file Table1.DOCX]

**Supplementary table 1.** MicroRNA expression at 2, 8 and 15 days after exposure to cadmium or TCDD, or ionizing radiation (at 1 or 5 Gy). Micro-RNAs showing a ≥1.5-fold change in their expression are indicated by bold font.
